# Trends in Mortality Among Females in the United States, 1900–2010: Progress and Challenges

**DOI:** 10.5888/pcd15.170284

**Published:** 2018-03-08

**Authors:** Robert A. Hahn, Man-Huei Chang, R. Gibson Parrish, Steven M. Teutsch, Wanda K. Jones

**Affiliations:** 1 Community Guide Branch, Office of Public Health Scientific Services, Centers for Disease Control and Prevention, Atlanta, Georgia; 2 Office of the Director, National Center for HIV/AIDS, Viral Hepatitis, STD, and TB Prevention, Centers for Disease Control and Prevention, Atlanta, Georgia.; 3 Senior Independent Consultant, Yarmouth, Maine; 4 Leonard D. Schaeffer Center for Health Policy and Economics, University of Southern California; Public Health Institute; UCLA Fielding School of Public Health; 5 Office of the Assistant Secretary for Health, US Department of Health and Human Services, Washington, DC

## Abstract

**Introduction:**

We analyzed trends in US female mortality rates by decade from 1900 through 2010, assessed age and racial differences, and proposed explanations and considered implications.

**Methods:**

We conducted a descriptive study of trends in mortality rates from major causes of death for females in the United States from 1900 through 2010. We analyzed all-cause unadjusted death rates (UDRs) for males and females and for white and nonwhite males and females from 1900 through 2010. Data for blacks, distinct from other nonwhites, were available beginning in 1970 and are reported for this and following decades. We also computed age-adjusted all-cause death rates (AADRs) by the direct method using age-specific death rates and the 2000 US standard population. Data for the analysis of decadal trends in mortality rates were obtained from yearly tabulations of causes of death from published compilations and from public use computer data files.

**Results:**

In 1900, UDRs and AADRs were higher for nonwhites than whites and decreased more rapidly for nonwhite females than for white females. Reductions were highest among younger females and lowest among older females. Rates for infectious diseases decreased the most. AADRs for heart disease increased 96.5% in the first 5 decades, then declined by 70.6%. AADRs for cancer rose, then decreased. Stroke decreased steadily. Unintentional motor vehicle injury AADRs increased, leveled off, then decreased. Differences between white and nonwhite female all-cause AADRs almost disappeared during the study period (5.4 per 100,000); differences in white and black AADRs remained high (121.7 per 100,000).

**Conclusion:**

Improvements in social and environmental determinants of health probably account for decreased mortality rates among females in the early 20th century, partially offset by increased smoking. In the second half of the century, other public health and clinical measures contributed to reductions. The persistent prevalence of risk behaviors and underuse of preventive and medical services indicate opportunities for increased female longevity, particularly in racial minority populations.

## Introduction

From the beginning of the 20th century to 2010, the life expectancy at birth for females in the United States increased by more than 32 years (1) However, new causes of death have emerged with changes in technology and the built environment (eg, the automobile and highways), emerging infections (eg, HIV), and behavior (eg, cigarette smoking). We analyzed trends in mortality rates among females at each decade from 1900 through 2010, focusing on major causes of death, and examined differences by age and by race. Historical trends may indicate future trends, contributing factors, opportunities for intervention when interventions are known, and research needs when they are not.

## Methods

Data for the analysis of decadal trends in mortality rates were obtained from yearly tabulations of causes of death from published compilations and from public use computer data files. Data for 1900 through 1940 were taken from mortality information from death registration states, which included 10 states and the District of Columbia in 1900 (40.5% of the US population) and gradually expanded to include all 48 states and the District of Columbia by 1933 (2). For decennial mortality rates from 1940 through 1960, a compilation of mortality information was used (3). The Centers for Disease Control and Prevention’s (CDC’s) Compressed Mortality File 1968–1992 and WONDER data system (Wide-ranging Online Data for Epidemiologic Research; http://wonder.cdc.gov) were used for mortality counts and census denominators from 1970 through 2010. Two physician epidemiologists linked ICD (International Classification of Diseases) codes. Because data specific to the black population became available beginning in 1970, we summarized only all-cause trends for this population for the available period and concentrated on prior trends for “nonwhites,” a category that also includes other racial groups. Because data for age-specific rates by race became available beginning in 1920, the tabulation of AADRs began in this year (Appendices I and II). We used the term “females” in this study, because all ages were included in the analysis. We refer to “major causes of death” to distinguish from slightly different classifications of “leading causes of death” used by CDC’s National Center for Health Statistics.

We analyzed all-cause unadjusted death rates (UDRs) for males and females and for white and nonwhite males and females from 1900 through 2010 in decadal years to indicate mortality burden. We analyzed UDRs for black persons beginning in 1970 when the data were first made available. We also computed age-adjusted all-cause death rates (AADRs) by the direct method using age-specific death rates and the 2000 US standard population (4). We analyzed several trends in mortality rates among females only. We analyzed trends in all-cause, age-specific death rates by white/nonwhite race from 1900 through 2010 in decadal years, major causes of death in 1900 and 2010, trends in all-cause AADRs from 1900 to 2010, and trends in AADRs for specific causes of death from 1920 to 2010 by white/nonwhite race. We also analyzed trends in AADRs from 1900 through 2010 for selected chronic conditions (ie, heart disease, stroke, and cancers combined), selected infectious diseases (ie, influenza and pneumonia, tuberculosis, and enteritis and diarrhea combined), and unintentional injuries (ie, unintentional motor vehicle [UI-MV] and nonmotor vehicle injuries [UI-NMV] combined).

## Results

### All-cause UDR for females and males and whites and nonwhites, 1900–2010

From 1900 to 2010, the UDR among females in the United States decreased from 1,646.9 per 100,000 to 787.4 per 100,000, an overall decrease of 52.2% (Table 1). Among males, the UDR decreased from 1,791.1 per 100,000 in 1900 to 812.0 per 100,000 in 2010, an overall decrease of 54.7% (Table 1). The male UDR exceeded the female UDR in all decadal years except 2000; by 2010, the male excess had decreased to 24.6.

**Table 1:** All-Cause Death Rates and Differences, by Sex and Race, Death Registration States, 1900–1930, and United States, 1940–2010^a^

Death Rate	1900	1910	1920	1930	1940	1950	1960	1970	1980	1990	2000	2010
**All**												
Female	1,646.9	1,374.3	1,258.2	1,036.7	954.6	823.5	809.2	807.8	785.3	812.0	855.0	787.4
Male	1,791.1	1,556.4	1,338.1	1,225.3	1,197.4	1,106.1	1,104.5	1,090.3	976.9	918.4	853.0	812.0
Difference	144.2	182.1	79.9	188.6	242.8	282.6	295.3	282.5	191.6	106.4	−2.0	24.6
**Females**												
White	1,629.7	1,355.3	1,212.6	981.4	919.4	803.3	800.9	812.6	806.1	846.9	912.3	857.3
Nonwhite	2,443.8	2,102.8	1,754.2	1,528.0	1,256.2	993.5	872.6	775.3	660.6	634.2	604.9	524.4
Black	—	—	—	—	—	—	—	829.2	733.3	747.9	733.0	642.7
Difference, nonwhite vs white	814.1	747.5	541.6	546.6	336.8	190.2	71.7	−37.3	−145.5	−212.7	−307.4	−332.9
Difference, black vs white	—	—	—	—	—	—	—	16.6	−72.8	−99.0	179.3	214.6
**Males**												
White	1,774.8	1,537.2	1,298.1	1,169.6	1,162.2	1,089.5	1,098.5	1,086.7	983.3	930.9	887.8	866.1
Nonwhite	2,565.3	2,233.2	1,780.6	1,739.0	1,513.7	1,251.1	1,152.0	1,115.9	936.5	851.5	692.4	595.6
Black	—	—	—	—	—	—	—	1,186.6	1,034.1	1,008.0	834.1	725.4
Difference, nonwhite vs white	790.5	696.0	482.5	569.4	351.5	161.6	53.5	29.2	−46.8	–79.4	−195.4	−270.5
Difference, black vs white	—	—	—	—	—	—	—	99.9	50.8	77.1	−53.7	−140.7

Abbreviation: —, data not available.

a Death rates are per 100,000 population. See Appendix I for sources of rates.

Decreases in all-cause UDRs from 1900 to 2010 were higher among nonwhites than among whites for females and males (Table 1). UDRs decreased by 78.5% among nonwhite females and 47.4% among white females and by 76.8% among nonwhite males and 51.2% among white males. Among females, the UDR among nonwhites exceeded that among whites by 814.1 per 100,000 in 1900; nonwhite excess deaths decreased steadily, and beginning in 1970 the white female UDR exceeded the nonwhite female UDR. Among males, the all-cause UDR among nonwhites exceeded that among whites by 790.5 per 100,000 in 1900; this excess decreased steadily, and by 2010 the UDR among white males exceeded the rate of nonwhite males by 270.5 per 100,000.

From 1970 to 2010, death rates increased by 5.5% among white females and decreased by 22.5% among black females. Rates decreased by 20.3% among white males and by 38.9% among black males.

### All-cause AADR for females and males, whites and nonwhites, 1900–2010

From 1900 to 2010, the AADR among females decreased from 2,410.4 per 100,000 to 634.9 per 100,000, a decrease of 73.7% (Table 2). Among males, the AADR decreased from 2,630.8 per 100,000 in 1900 to 887.1 per 100,000 in 2010, a decrease of 66.3% (Table 2). The male AADR exceeded the female AADR in all decades, with the greatest excess in 1970 at 570.7 per 100,000; the male excess was higher in 2010 than in 1900.

**Table 2:** Age-Adjusted Death Rates and Differences, by Sex and Race, Death Registration States, 1900–1930, and United States, 1940–2010^a^

Age-Adjusted Rate	1900	1910	1920	1930	1940	1950	1960	1970	1980	1990	2000	2010
**All**												
Females	2,410.4	2,171.3	2,081.3	1,798.3	1,599.4	1,236.0	1,105.3	971.4	817.9	750.9	731.4	634.9
Males	2,630.8	2,459.9	2,213.2	2,088.8	1,976.0	1,674.2	1,609.0	1,542.1	1,348.1	1,202.8	1,053.8	887.1
Difference	220.4	288.6	131.9	290.5	376.6	438.2	503.7	570.7	530.2	451.9	322.4	252.2
**Females**												
White	2,394.0	2,154.0	2,025.9	1,726.6	1,550.4	1,198.0	1,074.4	944.0	796.1	728.8	715.3	630.8
Nonwhite	3,308.0	2,875.3	2,756.2	2,530.1	2,057.5	1,574.1	1,340.5	1,166.5	963.6	881.5	805.1	636.2
Black	—	—	—	—	—	—	—	1228.7	1,033.3	975.1	927.6	752.5
Difference, nonwhite vs white	914.0	721.3	730.3	803.5	507.1	376.1	266.1	222.5	167.5	152.7	89.8	5.4
Difference, black vs white	—	—	—	—	—	—	—	284.7	237.2	246.3	212.3	121.7
**Males**												
White	2,613.2	2,441.9	2,166.6	2,013.8	1,925.2	1,642.5	1,586.0	1,513.7	1,317.6	1,165.9	1,029.4	878.5
Nonwhite	3,576.5	3,087.5	2,748.4	2,845.5	2,458.9	1,949.5	1,777.6	1,754.5	1,569.4	1,443.4	1,186.7	911.0
Black	—	—	—	—	—	—	—	1,873.9	1,697.8	1,644.5	1,403.5	1,104.0
Difference, nonwhite vs white	963.3	645.6	581.8	831.7	533.7	307.0	191.6	240.8	251.8	277.5	157.3	32.5
Difference, black vs white	—	—	—	—	—	—	—	360.2	380.2	478.6	374.1	225.5

a Age-adjusted rates are per 100,000 US 2000 standard population. See Appendix I for sources of rates.

Decreases in all-cause AADRs were higher among nonwhites than among whites for females and males (Table 2). The AADR decreased by 80.8% among nonwhite females and by 73.7% among white females and by 74.5% among nonwhite males and 66.4% among white males. Among females, the AADR among nonwhites exceeded that among whites by 914.0 in 1900; the excess decreased steadily to 5.4 per 100,000 in 2010. Among males, the all-cause AADR among nonwhites exceeded that among whites by 963.3 per 100,000 in 1900; the excess decreased inconsistently, and by 2010 the difference was 32.5 per 100,000. In 1970, the AADR from all causes was 30.2% higher among black than among white females. The difference remained at approximately this level until 2010, then fell to 19.3%. During the same period, the excess AADR among black males changed little, from 23.8% to 25.7% (Table 2).

### Age-specific all-cause death rates among females, by white and nonwhite race, 1900–2010

Relative decreases in death rates decreased by increasing age (Table 3). The greatest decreases were among ages 1 to 4 years, a decrease of 98.8% among whites and 99.3% among nonwhites. Rates of decrease were greater among nonwhite females than among white females, except for females aged 85 years or older.

**Table 3:** Female Age-Specific Death Rates, Difference Between 1900 and 2010 Death Rates, and Relative Change in Death Rates From 1900 to 2010, by Race, Death Registration States, 1900–1930, and United States, 1940–2010^a^

Age/Race	1900	1910	1920	1930	1940	1950	1960	1970	1980	1990	2000	2010	Difference	% Change
**<1 y**														
All	14,541.2	11,762.1	8,067.3	6,073.8	4,774.3	2,854.6	2,321.3	1,863.7	1,141.7	855.7	663.4	564.0	13,977.2	−96.1
White	14,262.8	1,1524	7,612.4	5,602.3	4,359.4	2,566.8	2,007.7	1,614.6	962.5	690.0	550.5	488.0	13,774.8	−96.6
Nonwhite	29,945.4	22,142.8	13,107.5	9,787.6	7,736.9	4,749	4,067.1	3,169.4	1,944.1	1,480.7	1,062.5	794.3	29,151.1	−97.3
**1–4 y**														
All	1,912.3	1,335.3	945.7	523.3	267.0	126.7	98.4	75.4	54.7	41.0	28.7	23.3	1,889	−98.8
White	1,869.4	1,304.5	900.4	478.4	241.2	112.2	85.2	66.1	49.3	36.1	25.5	21.6	1,847.8	−98.8
Nonwhite	4,350	2662.3	1,418.7	868.9	442.8	230.3	174.4	123.3	79.5	59.9	39.8	28.5	4,321.5	−99.3
**5−14 y**														
All	387.5	285.1	247.4	153.2	89.1	48.9	37.3	31.8	24.2	19.3	15.0	11.1	376.4	−97.1
White	376.2	277.9	232.2	140.3	81.3	45.1	34.7	29.9	22.9	17.9	14.1	10.6	365.6	−97.2
Nonwhite	1,006	591.5	393.3	255.3	144.1	75.0	53.4	42.3	29.8	25.1	18.3	12.7	993.3	−98.7
**15−24 y**														
All	577.6	423.3	496.7	319.3	181.1	89.1	61.3	68.1	57.5	49.0	43.1	36.4	541.2	−93.7
White	562.8	405.9	431.6	253.7	139.7	71.5	54.9	61.6	55.5	45.9	41.1	36.2	526.6	−93.6
Nonwhite	1,119.6	1051	1,083.5	818.0	503.1	216.4	106.1	108.8	68.0	61.7	50.4	37.1	1,082.5	−96.7
**25−34 y**														
All	815.2	611.7	712.7	443.1	274.3	142.7	106.6	101.6	75.9	74.2	63.5	64.0	751.2	−92.1
White	806.1	593.7	652.7	362.6	217.6	112.8	85.0	84.1	65.4	61.5	55.1	61.4	744.7	−92.4
Nonwhite	1,173.3	1,159.5	1,346.8	1,108.1	740.0	390.4	260.0	215.7	135.7	133.1	94.9	72.2	1,101.1	−93.8
**35−44 y**														
All	975.2	790.4	799.8	614.9	452.2	290.3	229.4	231.1	159.3	137.9	143.2	128.9	846.3	−86.8
White	962.1	766.0	726.4	516.8	367.2	235.8	191.1	193.3	138.2	117.4	125.7	122.8	839.3	−87.2
Nonwhite	1,558	1,637.3	1603.0	1,526.3	1,174.6	754.0	547.3	490.5	288.3	243.0	217.5	149.7	1,408.3	−90.4
**45−54 y**														
All	1,417.7	1,207.2	1,170.2	1,062.3	860.7	641.5	526.7	517.2	412.9	342.7	312.5	311.4	1,106.3	−78.0
White	1,397.6	1,178.2	1,086.8	924.6	746.8	546.4	458.8	462.9	372.7	309.3	281.4	295.1	1,102.5	−78.9
Nonwhite	2,390.1	2,429.2	2,335.3	2,518.5	2,108.9	1,554.9	1,144.9	979.4	687.8	537.4	464.3	376.8	2,013.3	−84.2
**55−64 y**														
All	2,576	2,366	2,243.9	2,123.7	1,800.4	1,404.8	1,196.4	1,098.9	934.3	878.8	772.2	643.5	1,932.5	−75.0
White	2,551.7	2,337.1	2,171.3	1,993.2	1,684.4	1,293.8	1,078.9	1,014.9	876.2	822.7	730.9	617.8	1,933.9	−75.8
Nonwhite	4,212.7	3,822.9	3,579.6	4,142.8	3,318.3	2,763.0	2,409.7	1,886.9	1,423.1	1,251.5	1,006	765.4	3,447.3	−81.8
**65−74 y**														
All	5,358.3	5,240.5	5,046.3	4,675.8	4,222.2	3,333.2	2,871.8	2,579.7	2,144.7	1,991.2	1,921.2	1,527.5	3,830.8	−71.5
White	5,341.7	5,224.1	4,993.3	4,601.9	4,153.6	3,242.8	2,779.3	2,470.7	2,066.6	1,923.5	1,868.3	1,504.9	3,836.8	−71.8
Nonwhite	6,642.1	6,189.2	6,035.3	6,003.1	5,227.5	4,610.7	3,981.4	3,675.6	2,856.2	2,552.3	2,269.0	1,652.9	4,989.2	−75.1
**75−84 y**														
All	11,877.3	11,740.1	11,589	10,663.4	10,368.6	8,399.6	7,633.1	6,677.6	5,440.1	4,883.1	4,814.7	4,137.7	7,739.6	−65.2
White	11,885.1	11,780.6	11,639	10,755.8	10,482.6	8,481.5	7,696.6	6,698.7	5,401.7	4,839.1	4,785.3	4,165.4	7,719.7	−65.0
Nonwhite	11,315.8	9,391.2	10,639.1	9,144.8	8,413.7	7,064.7	6,708.4	6,392.6	5,863.3	5,313.8	5,071.8	3,951.8	7,364.0	−65.1
**≥85 y**														
All	25,517.4	24,599.8	24,466.8	22,138.0	22,759.1	19,194.7	19,008.4	1,5518	14,746.9	14,274.3	14,719.2	13,219.2	12,298.2	−48.2
White	25,674.8	24,814	24,695.1	22,514.1	23,495.3	19,679.5	19,477.7	15,980.2	14,979.6	14,400.6	14,890.7	13,419.3	12,255.5	−47.7
Nonwhite	19,582.7	17,787.3	22,121.6	18,716.1	1,5971.0	13,366.8	12,871.2	10,288.9	11,922.3	12,863.1	13,069.2	11,516.1	8,066.6	−41.2

a Rates are per 100,000 population. “Difference” is the difference between the 2010 rate and the 1900 rate (ie, 2010 rate − 1900 rate). “Change” is the % change from the 1900 rate to the 2010 rate (ie, [2010 rate − 1900 rate]/[1900 rate]). See Appendix I for sources of rates.

Although decreases in death rates among nonwhite females were continuous in all decades from 1900 through 2010, decreases did not continue for white females in recent decades. Among white females, death rates increased from 1960 to 1970 in females aged 15 to 24, 35 to 44, and 45 to 54 years; from 1990 to 2000 in females aged 35 to 44 years; and from 2000 to 2010 in females aged 25 to 34 and 45 to 54 years (data not shown).

### Major causes of death among females in 1900 and 2010

The 5 major causes of death for females in 1900 (46.3% of all deaths) were pneumonia and influenza (198.5 per 100,000), tuberculosis (187.8 per 100,000), enteritis and diarrhea (134.9 per 100,000), heart disease (133.7 per 100,000), and stroke (107.7 per 100,000). Of these causes, only heart disease and stroke were among the 5 major causes in 2010. In 2010, the 5 major causes (59.7% of all deaths) were heart disease (184.9 per 100,000), all cancers (168.2 per 100,000), stroke (49.1 per 100,000), chronic lower respiratory diseases (46.3 per 100,000), and UI-NMV (21.8 per 100,000). Direct comparison of ranked causes between the 1900 and 2010 was not possible, because data for one major cause in 2010 (ie, chronic lower respiratory diseases) were not available in 1900.

### Cause-specific AADR among females (per 100,000 population)

**Figure 1:**
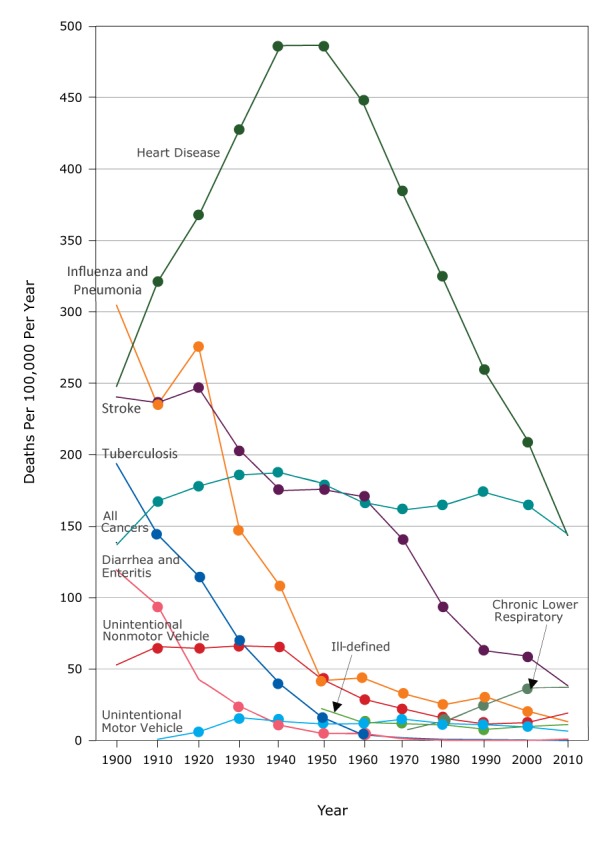
Age-adjusted death rates for major causes of death among all females, United States, 1900–2010. Abbreviation: —, data not available. **Table:** 

From 1900 to 2010, the greatest decreases in AADRs were among selected infectious diseases: the AADR for pneumonia and influenza decreased by 95.7%, tuberculosis by 99.9%, and enteritis and diarrhea by 99.2%; by 1950, rates of each of these conditions had already decreased by more than 85% (Table 4, Figure 1). AADRs for diseases of the heart increased by 96.5% in 1950 and then decreased rapidly by 2010 by 70.6%. AADR for stroke peaked in 1920 and subsequently decreased by 84.5%. AADRs for UI-NMV decreased by 63.9%.

**Table 4:** Female Age-Adjusted Death Rates for All Causes and Selected Causes, by Race and Available Years, 1900–2010^a^

Cause/Race	1900	1910	1920	1930	1940	1950	1960	1970	1980	1990	2000	2010
**All causes**												
All	2,410.4	21,71.3	2,081.3	1,798.3	1,599.4	1,236	1,105.3	971.4	817.9	750.9	731.4	634.9
White	2,394	2,154	2,025.9	1,726.6	1,550.4	1,198	1,074.4	944.0	796.1	728.8	715.3	630.8
Nonwhite	3,308	2,875.3	2,756.2	2,530.1	2,057.5	1,574.1	1,340.5	1,166.5	963.6	881.5	805.1	636.2
Black	—	—	—	—	—	—	—	1,228.7	1,033.3	975.1	927.6	752.5
**Selected infectious diseases^b^**												
All	617.8	473.5	434.1	241.2	157.2	63.3	52.5	35.6	25.8	31.1	20.9	14.1
White	—	—	412.1	220.7	142.3	55.5	48.7	33.8	25.5	30.9	20.8	14.0
Nonwhite	—	—	684.0	427.3	290.3	133.6	81.1	47.2	25.6	29.8	20.2	14.3
Black	—	—	—	—	—	—	—	48.5	26.1	30.9	21.4	15.0
**Influenza and pneumonia**												
All	304.7	234.9	276.1	147.4	107.6	41.9	43.8	32.7	25.1	30.5	20.7	13.1
White	—	—	269.0	141.3	102.1	38.8	41.2	31.4	25.0	30.5	20.7	13.0
Nonwhite	—	—	359.4	205.5	160.0	71.5	63.3	40.3	23.3	28.1	19.6	13.3
Black	—	—	—	—	—	—	—	41.6	23.9	29.1	20.8	14.0
**Tuberculosis**												
All	193.8	144.1	115.1	69.7	39.1	16.4	4.0	1.7	0.6	0.5	0.2	0.1
White	—	—	101.6	56.9	30.6	12.1	3.2	1.3	0.4	0.3	0.1	0.1
Nonwhite	—	—	265.3	183.8	111.7	54.0	11.2	5.1	2.2	1.6	0.6	0.3
Black	—	—	—	—	—	—	—	5.2	2.1	1.7	0.6	0.2
**Diarrhea and enteritis**												
All	119.3	94.5	42.9	24.1	10.5	5.0	4.7	1.2	0.1	0.1	0	0.9
White	—	—	41.5	22.5	9.6	4.6	4.3	1.1	0.1	0.1	0	0.9
Nonwhite	—	—	59.3	38.0	18.6	8.1	6.6	1.8	0.1	0.1	0	0.7
Black	—	—	—	—	—	—	—	1.7	0.1	0.1	0	0.8
**Selected chronic conditions^b^**												
All	626.6	726.8	793.9	819.1	850.9	844.7	786.4	684.8	579.4	495.4	437.6	328.3
White	—	—	792.5	811.9	847.2	830.9	774.4	674.7	570.3	485.4	429.8	324.5
Nonwhite	—	—	811.9	894.1	874.2	962.8	872.8	755.2	639.2	559.1	477.3	340.8
Black	—	—	—	—	—	—	—	798.3	687.9	617.4	551.7	405.7
**All cancers**												
All	138.6	169.5	180	188.1	189.6	182.3	168.7	163.2	166.7	175.7	167.6	146.7
White	—	—	181.9	190.7	191.3	182.0	167.7	162.5	165.2	174.0	166.9	146.9
Nonwhite	—	—	144.2	143.9	157.4	174.8	171.3	165.4	176.9	185.8	169.6	143.8
Black	—	—	—	—	—	—	—	173.4	189.5	205.9	197.9	170.8
**Cancers of respiratory system**												
All	—	—	—	—	—	6.9	7.6	14.0	25.3	38.1	42.1	38.8
White	—	—	—	—	—	7.0	7.5	13.9	25.3	38.5	43.0	39.9
Nonwhite	—	—	—	—	—	5.6	7.9	14.1	24.4	34.1	35.3	31.8
Black	—	—	—	—	—	—	—	14.7	26.1	38.2	41.1	37.5
**Major cardiovascular diseases**												
All	522.0	635.8	699.2	695.3	715.5	727.8	677.7	562.3	439.7	338.7	288.4	196.1
White	—	—	697.7	685.9	710.8	714.0	665.7	552.5	431.6	329.7	280.3	191.1
Nonwhite	—	—	736.3	812.8	764.8	852.9	766.4	631.0	492.3	396.9	333.1	216.7
Black	—	—	—	—	—	—	—	668.9	530.8	437.9	383.3	258.5
**Heart disease**												
All	247.6	320.9	367.0	427.8	486.4	486.6	447	381.6	320.8	257.09	210.9	143.3
White	—	—	365.2	423.5	486.9	479.2	441.7	376.7	315.9	250.9	205.6	140.4
Nonwhite	—	—	395.7	475.1	470.4	547.4	476.3	411.3	350.0	295.3	238.5	153.4
Black	—	—	—	—	—	—	—	435.6	378.6	327.5	277.6	185.3
**Stroke**												
All	240.4	236.4	246.9	203.2	174.9	175.8	170.7	140.0	91.9	62.7	59.1	38.3
White	—	—	245.4	197.7	169.0	169.7	165	135.5	89.2	60.5	57.3	37.2
Nonwhite	—	—	272	275.1	246.4	240.6	225.2	178.5	112.3	78.0	69.2	43.6
Black	—	—	—	—	—	—	—	189.3	119.8	84.0	76.2	49.6
**Chronic lower respiratory diseases**												
All	—	—	—	—	—	—	—	8.1	14.9	26.6	37.4	38.0
White	—	—	—	—	—	—	—	8.1	15.5	27.8	39.5	40.8
Nonwhite	—	—	—	—	—	—	—	6.9	8.6	15.5	20.3	19.3
Black	—	—	—	—	—	—	—	7.1	9.1	16.6	22.7	22.8
**Unintentional injuries^b^**												
All	52.9	66.5	70.5	82.3	78.8	54.5	40.7	36.9	27.2	22.5	22.0	25.6
White	—	—	71.3	84.0	80.2	54.4	39.7	36.0	26.7	22.2	22.2	27.3
Nonwhite	—	—	60.0	61.0	58.0	48.9	44.8	41.2	31.3	24.2	20.0	17.5
Black	—	—	—	—	—	—	—	41.7	31.9	25.3	21.3	19.3
**Unintentional motor vehicle injuries**												
All	—	0.8	6.0	16.0	13.4	11.5	11.7	14.9	11.8	11.0	9.5	6.5
White	—	—	6.3	16.4	13.6	11.4	11.7	14.9	12.2	11.2	9.8	6.8
Nonwhite	—	—	2.5	11.7	10.7	11.7	11.0	14.7	9.6	10.1	8.5	5.6
Black	—	—	—	—	—	—	—	14.1	8.5	9.6	8.4	5.9
**Unintentional nonmotor vehicle injuries**												
All	52.9	65.7	64.5	66.3	65.4	43.0	29.0	22.0	15.4	11.5	12.5	19.1
White	—	—	65.0	67.6	66.6	43.0	28.0	21.1	14.5	11.0	12.4	20.5
Nonwhite	—	—	57.5	49.3	47.3	37.2	33.8	26.5	21.7	14.1	11.5	11.9
Black	—	—	—	—	—	—	—	27.5	23.4	15.7	12.9	13.4
**Ill-defined conditions**												
All	—	—	—	—	—	22.2	12.7	11.6	10.8	7.7	9.7	11.1
White	—	—	—	—	—	16.9	9.3	8.6	8.9	6.8	9.3	11.0
Nonwhite	—	—	—	—	—	84.1	48.9	39.3	27.2	13.0	11.3	10.7
Black	—	—	—	—	—	—	—	42.0	29.6	14.9	13.6	13.4
**Senility**												
All	—	—	—	—	—	—	—	1.3	0.8	0.7	1.5	1.8
White	—	—	—	—	—	—	—	1.1	0.7	0.7	1.5	1.9
Nonwhite	—	—	—	—	—	—	—	3.7	1.9	0.9	1.3	1.3
Black								3.8	1.8	1.0	1.5	1.5
**Other ill-defined conditions**												
All	—	—	—	—	—	—	—	10.2	10.0	7.0	8.2	9.3
White	—	—	—	—	—	—	—	7.4	8.1	6.1	7.8	9.1
Nonwhite	—	—	—	—	—	—	—	35.2	25.1	20.7	10.0	9.4
Black	—	—	—	—	—	—	—	38.2	27.8	13.9	12.1	11.9

Abbreviation: —, data not available.

a Age-adjusted rates are per 100,000 US 2000 standard population. See Appendix I for sources of rates.

b Selected infectious disease totals are the sum of the rates for influenza and pneumonia, tuberculosis, and enteritis and diarrhea. Selected chronic condition totals are the sum of the rates for heart disease, stroke, and all cancers. They do not include chronic lower respiratory diseases. Unintentional injury totals are the sum of the rates for unintentional motor vehicle and nonmotor vehicle injuries.

Cause	1900	1910	1920	1930	1940	1950	1960	1970	1980	1990	2000	2010
Influenza and pneumonia	304.7	234.9	276.1	147.4	107.6	41.9	43.8	32.7	25.1	30.5	20.7	13.1
Tuberculosis	193.8	144.1	115.1	69.7	39.1	16.4	4.0	1.7	0.6	0.5	0.2	0.1
Diarrhea and enteritis	119.3	94.5	42.9	24.1	10.5	5.0	4.7	1.2	0.1	0.1	0	0.9
All cancers	138.6	169.5	180.0	188.1	189.6	182.3	168.7	163.2	166.7	175.7	167.6	146.7
Heart disease	247.6	320.9	367.0	427.8	486.4	486.6	447.0	381.6	320.8	257.0	210.9	143.3
Stroke	240.4	236.4	246.9	203.2	174.9	175.8	170.7	140.0	91.9	62.7	59.1	38.3
Chronic lower respiratory	—	—	—	—	—	—	—	8.1	14.9	26.6	37.4	38.0
Unintentional motor vehicle injuries	—	0.8	6.0	16.0	13.4	11.5	11.7	14.9	11.8	11.0	9.5	6.5
Unintentional nonmotor vehicle injuries	52.9	65.7	64.5	66.3	65.4	43.0	29.0	22.0	15.4	11.5	12.5	19.1
Ill-defined conditions	—	—	—	—	—	22.2	12.7	11.6	10.8	7.7	9.7	11.1

The AADR for 3 causes of death increased greatly during the study period (Table 4). This was in contrast to the AADR for all cancers, which increased to a peak in 1940 and decreased by 22.6% thereafter. The AADR for cancers of the respiratory system (including lung cancer) was 6.9 per 100,000 in 1950 and increased to 38.8 per 100,000 in 2010. Chronic lower respiratory conditions, first reported in 1970 at 8.1 per 100,000, increased by 369.1% to 38.0 per 100,000 in 2010. The AADR for motor-vehicle injury-related deaths, first recorded in 1910 at 0.8 per 100,000, peaked at 16.0 per 100,000 in 1930 and subsequently decreased by 59.4%.

### AADR among white females compared with nonwhite females, 1920–2010

From 1920 through 2010, the decrease in AADR from all causes was greater among nonwhite females (76.9%) than among white females (68.9%) (Table 4). From 1920 through 2010, AADRs reached a peak one decade earlier for whites than for nonwhites for heart disease (1940 and 1950, respectively) and stroke (1920 and 1930, respectively), and subsequently decreased. AADRs decreased similarly for white and nonwhite females for tuberculosis, enteritis and diarrhea, and pneumonia and influenza; were slightly greater for UI-NMV deaths for nonwhite females than for white females; and were substantially greater for whites than for nonwhites for cancers (Table 4). The increase of UI-MV deaths was far greater for nonwhite females than for white females.

### AADR trends among females for major selected chronic, infectious, and unintentional injury conditions, 1900–2010

**Figure 2:**
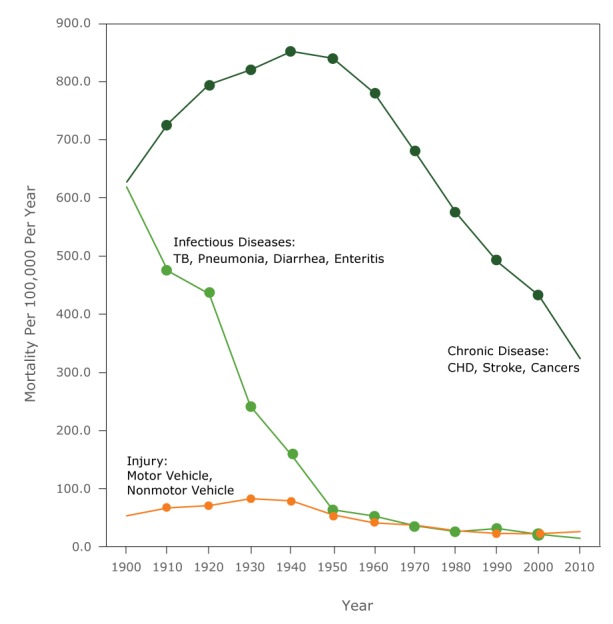
Age-adjusted death rates for chronic, infectious disease, unintentional injury among all females, United States, 1900–2010. Selected chronic conditions include heart disease, stroke, and cancers combined; selected infectious diseases include influenza and pneumonia, TB, and enteritis and diarrhea combined; unintentional injuries includes unintentional motor vehicle and nonmotor vehicle injuries combined. Abbreviations: CHD, coronary heart disease; TB, tuberculosis. **Table:** 

In 1900, the selected chronic, infectious, and injury conditions assessed in this analysis accounted for 53.8% of the female AADR (Table 4, Figure 2). Trends in the AADR for these selected conditions varied markedly. Rates for selected chronic conditions increased to a peak in 1940 and 1950, then decreased. Rates for the infectious conditions decreased rapidly, began to level out in 1950, and decreased slowly thereafter. Unintentional injury rates rose slightly to a maximum in 1930, declining slowly thereafter.

Cause	1900	1910	1920	1930	1940	1950	1960	1970	1980	1990	2000	2010
Selected chronic conditions	626.6	726.8	793.9	819.1	850.9	839.0	779.7	678.0	573.0	489.2	431.7	323.1
Selected infectious diseases	617.8	473.5	434.1	241.2	157.2	63.3	52.5	35.6	25.8	31.1	20.9	14.1
Unintentional injuries	52.9	66.5	70.5	82.3	78.8	54.5	40.7	36.9	27.2	22.5	22.0	25.6

## Discussion

During the past 110 years, mortality rates among females in the United States decreased substantially, particularly for younger females and nonwhite females. Mortality rates from infectious diseases decreased precipitously by mid-century. In contrast, heart disease mortality rates among females increased in the first half of the century; they then decreased during the second half. Mortality from respiratory system cancers and chronic respiratory disease among females also rose dramatically, starting in the mid-1960s. The early risk in UI-MV mortality quickly plateaued and then decreased.

Providing explanations for observed trends is challenging (Appendix III). Available data allow only ecological and retrospective analyses. Sources vary in accuracy, specificity, and consistency over time. Exposures commonly interact, but information on conjoint exposures is lacking. Racial categories and disease categories changed during the study period. Explanations are thus hypothetical and imprecise.

We divided potential explanations into social and environmental factors, such as education, employment, poverty, sanitation, housing, and transportation; biological and behavioral factors, such as hypertension, cholesterol levels, cigarette smoking, physical activity, and diet; and preventive and therapeutic interventions, such as measles vaccination, hypertension screening, and treatment of cardiovascular disease. Changes in ICD coding may also account for some trends observed. Overall, social and environmental explanations and biological and behavioral explanations predominate in the first half of the 20th century, whereas preventive and therapeutic interventions then gained importance.

Twenty years of the 30-year increase in female life expectancy from 1900 to 2010 occurred between 1900 and 1950 (1), affected principally by social and environmental factors. During the first half of the 20th century, sanitation improved substantially, with greater benefits for blacks than for whites (5,6). Sanitation, the provision of clean drinking water and safe disposal of sewage and solid waste, affected rates of infectious and chronic diseases (6) and was associated with almost half the total decrease in mortality rates in major US cities between 1900 and 1940, three-quarters of the decrease in infant mortality rates, and almost two-thirds of the decrease in child mortality rates (5).

Decreases in infectious disease mortality rates were also probably associated with improved housing, increased education and income, and reduced poverty (7). From 1900 to 1940 the mean number of occupants per housing unit in the United States fell from 4.8 to 3.8, reducing infectious disease contagion (8). The large decrease in major infectious diseases preceded deployment of antibiotics and immunization.

Education, an established determinant of health, increased substantially during this period. From 1900 to 1950, female high school graduation rates increased from almost 30% to approximately 80% (9). Postsecondary education for females also increased (10). Between 1940 and 1970, the median years of schooling increased for nonwhite and white females (8).

There were increases in proportions of the workforce that was female, of married females working outside the home, of female compared with male earnings, and of black compared with white earnings (10), all contributing to improved economic well-being for females and nonwhites and likely to have led to improved health.

Three major nonexclusive explanations for increased heart disease mortality rates are possible. First, as understanding of diseases improved, the apparent rise may have partly resulted from changes in classifying and assigning causes of death during the first half of the century. Examples include the shift from nonspecific causes, (eg, “ill-defined conditions”) to specific causes (eg, ischemic heart disease) and the 1929 reclassification of diseases of the coronary arteries from “diseases of the arteries” to a new subcategory of heart disease, “diseases of the coronary arteries and angina pectoris” (11,12). Second, the rise has also been attributed to a reduction in “competing causes” of death, most notably the reduction of deaths due to infectious and diarrheal diseases (13).

Third, cigarette smoking was a major influence on trends in female chronic disease mortality rates, particularly trends for heart disease, respiratory system cancers, and chronic lower respiratory disease (14–16). The prevalence of cigarette smoking among females rose rapidly in the 1930s, peaked from about 1965 to 1975, and decreased thereafter (15–18). This trend is consistent with the rise of cancer death rates among females in the 1960s. Smoking is also associated with particularly high relative risks for heart disease and stroke among females younger than 50 (15,16). It is plausible that the rapid rise of smoking in the first half of the century was also associated with much mortality, principally from heart disease (19). Holford et al estimated that the decrease of smoking among females between 1964 and 2012 averted 2.7 million deaths from all causes (19). However, the decrease in deaths predated the decrease in smoking among females by 5 to 10 years, suggesting that other factors contributed (13,20). Investigators have called for additional research to explain the 20th century rise and decrease of heart disease (13,20).

Other causes of heart disease and of stroke merit assessment. Estimates of blood pressure trends indicate that blood pressures decreased during the 20th century (21), which is consistent with the decrease of stroke mortality rates during the study period. Nationally representative estimates of rates of hypertension in 1960 were 26.4% among white females and 43.1% among black females (22). Between 1988 and 1991, rates had fallen to 16.7% and 28.1%, respectively (22). Control of hypertension has increased (23). Trends in hypertension control are consistent with the accelerating decrease in trends in stroke deaths after 1970.

Nationally, the mean number of kilocalories consumed (ie, acquired but not necessarily ingested) per capita per day (for males and females combined) increased from approximately 3,400 from 1909 to 1919 to 3,900 in 2000 (24). Nationally representative estimates of rates of elevated cholesterol blood levels — another risk factor for heart disease — are not available before 1960, after which mean levels among both black and white females slowly decreased (25). Criteria for overweight have also changed since 1960, but trends indicate a substantial rise for both black and white females (26). The prevalence of diabetes in the population — another risk factor for heart disease — rose from 1% in 1958 to 7% in 2011 (data not available for females alone) (27). During the 20th century, total physical activity decreased — a protective factor for heart disease and stroke (28). Long-term trends in overweight, physical activity, and the prevalence of diabetes do not explain the trends in heart disease and stroke without considering the effects of improved medical care (26–28). The decrease in saturated fat ingestion is consistent with declining heart disease and stroke mortality rates in the second half of the 20th century (25).

In the last half of the 20th century clinical interventions were developed and deployed for the treatment of heart disease and stroke (29). Ford and colleagues estimated that 47% of the decrease in heart disease mortality rates since 1980 are attributable to clinical treatments and 44% to changes in risk factor prevalence (30). Conversely, increases in the prevalence of overweight and diabetes may have slowed the rate of decrease in heart disease mortality rates. Various screening tests (eg, mammography, Pap tests, colonoscopy) also affect mortality associated with specific cancers. Their use in the second half of the 20th century, along with advances in treatment, may be responsible for some of the decrease of age-adjusted cancer mortality rates (31).

Since 1998, the anomalous increasing death rate among white females in certain age groups has been noted, particularly among those with a high school education or less. Deaths are largely attributed to drug and alcohol poisoning, suicide, chronic liver disease, and cirrhosis (32). We found that these increases began earlier than previously reported and affected additional age groups.

The lack of large increases in UI-MV deaths during the 20th century is notable (33). From 1900 to 1970, the number of automobiles rose from 4,100 to 6.5 million; from 1923 to 1970, the miles of federally supported highways grew five fold; miles driven per capita annually climbed almost six fold from 1930 to 2000; and from 1945 to 1970, average vehicle highway speed increased from 45 to 60 miles per hour (8). Large increased exposure to risks for UI-MV deaths, is not, however, reflected in long-term trends in UI-MV mortality rates. Deaths per mile driven reached a low peak in 1930 and gradually decreased thereafter, probably in association with safety measures (34). In the first 3 decades of the 20th century, many UI-NMV deaths were from falls, drowning, railroad injuries, and burns (2); these causes decreased greatly after 1940. However, in recent decades deaths among females from drug overdoses have risen rapidly and since 2007 have exceeded deaths from UI-MV.

Much of the decrease in mortality rates among females in the past 110 years is attributable to improvements in major social and environmental determinants of health — education, income, housing, and sanitation. The rapid decrease in mortality rates from infectious by mid-century largely preceded the widespread use of antibiotics or immunization. The extent and specific causes of increased heart disease mortality rates among females in the first half of the century remain uncertain. The decrease of heart disease mortality rates during the second half of the century may be the result of multiple factors. The dramatic rise in mortality rates from respiratory system cancers and chronic respiratory disease among females is most likely due to cigarette smoking. The plateau in UI-MV mortality rates, despite the rapid growth of automobile use during the century, is probably a result of early safety measures.

Trends in mortality rates during the past century reflect major patterns of health determinants. Sanitary and safety improvements along with understanding of and therapies for infectious diseases led to great reductions in infectious causes of death. With increasing longevity and more sedentary lifestyles, chronic diseases increased as major causes of death. Although some of these causes, particularly heart disease and stroke, decreased as a result of behavior change and effective health care (22), decreases in mortality rates are slowing. Ongoing and expanded efforts to control underlying determinants should accelerate decreases in mortality rates and reduce inequities.

## Appendix I Data sources and International Classification of Diseases (ICD) Codes Used for Death Rates, 1900–2010

**Table:**

Cause and Rate	Data Years and ICD Revision								
	1900 (ICD 1)	1910, 1920 (ICD 2)	1930 (ICD 4)	1940 (ICD 5)	1950 (ICD 6)	1960 (ICD 7)	1970 (ICD 8)	1980, 1990 (ICD 9)	2000, 2010 (ICD 10)
All causes									
**DR**	Hist290	Hist290	Hist290	Hist290	Hist290	Hist290	Hist290	Hist290	Wonder, NCHS 2013
**AADR**	Hist293	Hist293	Hist293	Hist293	Hist293	Hist293	Hist293	Hist293	Wonder, NCHS 2013
Influenza and pneumonia	10, 92, 93	10, 91, 92	11, 107–109	33, 107–109	480–493	480–493	470–474, 480–486	480–487	J10–J18
**DR**	Hist290	Hist290	Hist290	Hist290	Hist290	Hist290	Hist290	Hist290	Wonder, NCHS 2013
**AADR**	Hist293	Hist293	Hist293	Hist293	Hist293	Hist293	Hist293	Hist293	Wonder, NCHS 2013
Tuberculosis	26–34, 35	28–35	23–32	13–22	001–019	001–019	010–019	010–018	A16–A19
**DR**	Hist290	Hist290	Hist290	Hist290	Hist290	Hist290	Hist290	Hist290	Wonder, NCHS 2013
**AADR**	Hist293	Hist293	Hist293	Hist293	Hist293	Hist293	Hist293	Hist293	Wonder, NCHS 2013
Diarrhea and enteritis	13, 105, 106	13, 104, 105	119, 120	119, 120	543, 571, 572	543, 571, 572	009	009	A09
**DR**	Hist290	Hist290	Hist290	Hist290	Hist290	Hist290	Wonder	Wonder	Wonder
**AADR**	Hist293	Hist293	Hist293	Hist293	Hist293	Hist293	Wonder	Wonder	Wonder
Cancers (malignant neoplasms)^a^	39–45	39–45	45–53	45–55	140–205	140–205	140–209	140–208	C00– C97
**DR**	Linder T15	Linder T15	Linder T15	Linder T15	Hist290	Hist290	Hist290	Hist290	Wonder, NCHS 2013
**AADR**	Hist293	Hist293	Hist293	Hist293	Hist293	Hist293	Hist293	Hist293	Wonder, NCHS 2013
Cancers of respiratory system	NA	NA	47	47	160–164	160–164	160–163	160–165	C30–C39
**DR**	NA	NA	NA	Grove T63	Hist290	Hist290	Hist290	Hist290	Wonder
**AADR**	NA	NA	NA	NA	Hist293	Hist293	Hist293	Hist293	Wonder
Major cardiovascular diseases^a^	64–66, 77–86, 142	47, 64–66, 77–85, 142	56, 82, 90–95, 97–103	58, 83, 90–103	330–334, 400–468	330–334, 400–468	390–448	390–448	I00–I78
**DR**	Hist290	Hist290	Hist290	Grove T63	Hist290	Hist290	Hist290	Hist290	Wonder, NCHS 2013^,^
**AADR**	Hist293	Hist293	Hist293	Hist293	Hist293	Hist293	Hist293	Hist293	Wonder, NCHS 2013
Heart diseases^a^	77–80	77–80	90–95	90–95	400–402, 410–443	400–402, 410–443	390–398, 402, 404, 410–429	390–398, 402, 404, 405–409, 410–429	I00–I09, I11, I13, I20–I51
**DR**	Hist290	Hist290	Hist290	Hist290	Hist290	Hist290	Hist290	Hist290	Wonder, NCHS 2013
**AADR**	Hist293	Hist293	Hist293	Hist293	Hist293	Hist293	Hist293	Hist293	Wonder, NCHS 2013
Stroke (cerebrovascular diseases)	64–66, 82	64–66, 82	82	83	330–334	330–334	430–438	430–438	I60–I69
**DR**	Hist290	Hist290	Hist290	Hist290	Hist290	Hist290	Hist290	Hist290	Wonder, NCHS 2013
**AADR**	Hist293	Hist293	Hist293	Hist293	Hist293	Hist293	Hist293	Hist293	Wonder, NCHS 2013
Chronic lower respiratory diseases	NA	NA	NA	NA	NA	241, 501, 502, 527.1	490–493, 519.3	490–496	J40–J47
**DR**	NA	NA	NA	NA	NA	Hist290	Hist290	Hist290	Wonder, NCHS 2013
**AADR**	NA	NA	NA	NA	NA	NA	Wonder	Hist293	Wonder, NCHS 2013
Unintentional injuries (accidents)	164–175, 176pt	165–181, 185, 186	176, 178–195, 201–214	169–176, 178–195	E800–E962	E800‐E962	E800–E949	E800–E949	V01–X59, Y85–Y86
**DR**	Hist290	Hist290	Hist290	Hist290	Hist290	Hist290	Hist290	Hist290	Wonder, NCHS 2013
**AADR**	Hist293	Hist293	Hist293	Hist293	Hist293	Hist293	Hist293	Hist293	Wonder, NCHS 2013
Unintentional motor vehicle injuries (motor vehicle accidents)	166part	175part	206, 208, 210, 211	170	E810–E835	E810–E835	E810–E823	E810–E825	V02–V04, V09.0, V09.2, V12–V14, V19.0–V19.2, V19.4–V19.6, V20–V79, V80.3–V80.5, V81.0–V81.1, V82.0–V82.1, V83–V86, V87.0– V87.8, V88.0–V88.8, V89.0, V89.2
**DR**	Hist290	Hist290	Hist290	Hist290	Hist290	Hist290	Hist290	Hist290	Wonder, NCHS 2013
**AADR**	Hist293	Hist293	Hist293	Hist293	Hist293	Hist293	Hist293	Hist293	Wonder, NCHS 2013
Unintentional injuries, nonmotor vehicle	164, 165, 166pt, 167–175, 176pt	165–174, 175pt, 176–181, 185, 186	176, 178–195, 201–205, 207, 209, 212–214	169, 171–176, 178–195	E800–E802, E840–E962	E800–E802, E840–E962	E800–E807, E825–E949	E800–E807, E826–E949	V01, V05–V06, V09.1, V09.3–V09.9, V10–V11, V15–V18, V19.3, V19.8–V19.9, V80.0–V80.2, V80.6–V80.9, V81.2–V81.9, V82.2–V82.9, V87.9, V88.9, V89.1, V89.3, V89.9, V90–V99, W00–X59, Y85–Y86
**DR**	Hist290	Hist290	Hist290	Hist290	Hist290	Hist290	Hist290	Hist290	Wonder, NCHS 2013
**AADR**	Hist293	Hist293	Hist293	Hist293	Hist293	Hist293	Hist293	Hist293	Wonder, NCHS 2013
Ill-defined conditions^a^	154, 177–179	154, 187–189	162, 199, 200	162, 199, 200	780–795	780–795	780–796	780–799	R00–R99
**DR**	Linder T15	Linder T15	Linder T15	Hist290	Hist290	Hist290	Hist290	Hist290	Wonder, NCHS 2013
**AADR**	NA	NA	NA	NA	Hist293	Hist293	Hist293	Hist293	Wonder, NCHS 2013
Senility	154	154	162	162	794	794	794	797	R54
**DR**	Linder T15	Linder T15	Linder T15	Linder T15	Grove T65	Grove T65	Wonder	Wonder	Wonder
**AADR**	NA	NA	NA	NA	NA	NA	Wonder	Wonder	Wonder
Other ill-defined conditions	177–179	187–189	199, 200	199, 200	780–793, 795	780–793, 795	780–793, 795–796	780–796, 798–799	R00–R53, R55–R99
**DR**	Linder T15	Linder T15	Linder T15	Linder T15	NA	NA	Wonder	Wonder	Wonder
**AADR**	NA	NA	NA	NA	NA	NA	Wonder	Wonder	Wonder

Abbreviations: AADR, age-adjusted death rate; DR, death rate; NCHS, National Center for Health Statistics.

**^a^** Hist290 and Hist293 rates for 1900 for major cardiovascular diseases do not include ICD 1 code 47. From 1900 through 1940, leukemia was not classified with cancers, and rates for leukemia are only available for these years for the whole population (ie, without sex- or race-specific rates). Thus, for 1900 through 1940, cancer rates do not include deaths from leukemia. Hist290 Death Rates for 1970 for heart disease do not include ICD 8 code 402. The title for “Ill-defined Conditions” has varied among the 10 ICD revisions. From 1900 through 1940, senility was classified separately and not as an “Ill-defined Condtion.” From 1950 through 2010, senility was classified within Ill-defined Conditons (ICD-6 through ICD-9) or within Symptoms, signs and abnormal clinical and laboratory findings, not elsewhere classified (ICD-10).

## Appendix II Sources of Mortality Data.

Data for the analysis of decadal mortality trends were obtained from yearly tabulations of causes of death from published compilations and from public use computer data files. Data for 1900 through 1940 were drawn from mortality information from death registration states, which included 10 (out of 45) states and the District of Columbia in 1900 (representing 40.5% of the U.S. population) and gradually expanded to include all 48 states and the District of Columbia by 1933 (1). For decennial mortality rates from 1940 through 1960, a compilation of mortality information was utilized (2). CDC’s Compressed Mortality File 1968–1992 and WONDER data system (Wide-ranging Online Data for Epidemiologic Research; http://wonder.cdc.gov) were used for mortality counts and census denominators from 1970 through 2010 (3). Two physician epidemiologists (S.M.T. and R.G.P.) linked ICD codes (Appendix I).

To the extent possible, we focused on the 5 causes of death that were major causes either in 1900 or 2010, using standard International Classification of Diseases (ICD) condition classifications and available data.We use the the term “major” to distinguish causes examined here from the “leading” used by the CDC National Center for Health Statistics. Causes of death were coded according to the particular revision of the ICD in use, starting in 1900. The ICD underwent 10 revisions during the 110-year period analyzed. Available sources link succeeding ICD codes and estimate differential counts associated with ICD changes (4,5). Several ICD changes substantially distort observed trends: apparent declines in pneumonia and influenza between 1900 and 2000 are substantially affected by revisions in ICD coding (6). The trend in stroke before and after 1950 is substantially affected by the revision in ICD 6. The trend in chronic lower respiratory disease beginning in 1970 has also been substantially affected by changes in ICD coding.

Beginning with the ICD 6 in 1949, the “underlying cause of death” in sources was selected by standardized algorithms from the medical conditions on the death certificate. From 1900 through 1940, leukemia was not classified with cancers, and rates for leukemia are only available for these years for the whole population (ie, without sex- or race-specific rates). In order to show rates for cancers for the entire period 1900–2010, leukemia is excluded from the “all cancer” category for 1900–1940 in this report but is included thereafter. Because unintentional non motor vehicle injury (UI-NMV) mortality (ie, deaths from injury sources other than motor vehicles) becomes the fifth major cause in 2010, we also report trends in motor (UI-MV) vehicle mortality; although not currently among the major 5 causes, UI-MV mortality was the fourth or fifth major cause from 1970–1990.

Because data specific to blacks became available only beginning in 1970, we summarized only all-cause trends for blacks for the available period and concentrated on trends over the century for “nonwhites,” a category that also includes racial groups other than blacks. In 1970, black females constituted 89.2% of the nonwhite female population (http://wonder.cdc.gov/welcomeT.html). By 2010, the proportion had declined to 66.7%; thus the “nonwhite” category has become increasingly unrepresentative of the black population. Mortality data on Hispanics became available only after 1980 and are thus not reported in this longterm trend analysis.

References:

1. Linder F, Grove R. Vital statistics rates in the United States, 1900–1940: US Government Printing Office; 1943.

2. Grove RD, Hetzel AM. Vital statistics rates in the United States 1940–1960. US Department Of Health, Education, and Welfare; Public Health Service; National Center for Health Statistics. Washington (DC): US Government Printing Office; 1968. Table 63: Death Rates for 35 selected causes by age, color, and sex: United States, 1940–60. http://www.cdc.gov/nchs/data/vsus/vsrates1940_60.pdf. Accessed January 13, 2018.

3. CDC WONDER: Centers for Disease Control and Prevention, National Center for Health Statistics. CDC WONDER Online Database. http://wonder.cdc.gov/.

4. Faust M, Dolman A. Comparability ratios based on mortality statistics for the fifth and sixth revisions, United States, 1950. Washington (DC): US Department of Health, Education, and Welfare, Public Health Service, National Center for Health Statistics; 1964.

5. Klebba A, Scott JH. Estimates of selected comparability ratios based on dual coding of 1976 death certificates by the Eighth and Ninth Revisions of the International Classification of Diseases. Monthly Vital Statistics Report 1980;28(11).

6. Anderson RN, Miniño AM, Hoyert DL, Rosenberg HM. Comparability of cause of death between ICD-9 and ICD-10: preliminary estimates. National vital statistics reports 2001;49(2):1–32.

## Appendix III Study Limitations.

Mortality is not an optimal measure of the burden of disease and suffering and thus is not ideal for planning and monitoring interventions to improve public health. However, mortality is the only measure available over the time span examined. Measures combining morbidity and estimates of suffering or lost ability, such as healthy life expectancy (HLE) or disability adjusted life-years (DALYs), are only available for recent decades. In addition, available mortality data are imperfect:

1. Data on all states in the United States became available only in 1933, as all states joined in the federal death registration area (1). Thus, early rates may not be representative of the US population and early trends are assessed in different populations over time.

2. Analysis of mortality rates is limited by the consistency and completeness of data available, as noted by Linder and Grove (http://www.cdc.gov/nchs/data/vsus/vsrates1900_40.pdf). Completeness of population and mortality reporting is likely to have increased substantially since 1900. Incomplete reporting leads to underestimates of death rates. The completeness of death reporting (ie, assurance that all deaths that occur are reported) has not been assessed; however, participation in the death registration system required that >90% of deaths were reported. It is believed that death reporting is more complete than birth reporting which, in 1940 and 1950, was 92.5% and 97.9%, respectively, though variable among states (2).

3. With the growth of diagnostic and clinical knowledge, disease classifications changed substantially over time, and the linkage of diagnostic categories across coding systems is difficult. For example, leukemias were not classified with other cancers until 1940. (However, leukemias currently constitute approximately 3.9% of female cancer mortality [3] so that this exclusion does not greatly affect the assessment of cancer trends.)

4. The clear mortality effect modification by age which we report suggests not reporting mortality rates for all ages combined. However, the paper would have been inordinately complex and long had we stratified the analysis by age.

5. With the exception of 1910 and 1920 both of which used ICD-2, 1980 and 1990 both of which used the ICD-9, and 2000 and 2010 both of which used the ICD-10, each decade for which mortality rates are reported in this analysis used a different ICD classification system for cause-of-death (Appendix I). Data were not available for all conditions throughout the period examined, sometimes because they were not relevant (eg, automobile-related injury prior to the expansion of automobile travel and chronic respiratory disease prior to widespread cigarette smoking) and sometimes because of lack of understanding.

6. The comparability of ICD categories between revisions is commonly assessed by the comparability ratio, which compares the number of deaths attributed to a given cause using the previous ICD revision to the number of deaths in the same year attributed to the comparable cause for the subsequent ICD revision (4). For example, comparability ratios among 56 causes for the 6th and 5th ICD range from 0.57 (for “sepsis of pregnancy, childbirth, and puerperium”) to 1.79 (for “acute nephritis and nephritis with edema, including nephrosis”); there were 10 causes with comparability ratios between 0.99 and 1.01 (4).

7. Decedents may have several diagnoses at the time of death. The method of selecting the underlying cause from among causes listed on death certificates changed during the study period. While in the fifth ICD, tabulated mortality information was based on a rule for selecting a single cause from all causes listed, in the sixth and seventh ICDs, the underlying cause was that reported by the certifying physician, unless this selection was clearly implausible. This change is known to have affected reported rates of diabetes and nephritis as causes of death (5). There is also evidence that coronary heart disease is often over-diagnosed (6), which may inflate CHD disease rates and, if overdiagnosis has changed over time, make apparent trends inaccurate.

8. The nosological category, “ill-defined causes,” is problematic. “Ill-defined conditions” is an explicitly residual category reflecting the state of knowledge at the time of diagnosis (http://www.theodora.com/diseases/ill_defined_conditions.html). In 1900, this category accounted for 4.0% of deaths. This proportion declined to 1.5% in 2010, suggesting an improvement in diagnostic specificity. From 1970 to 2010, ill-defined causes declined more rapidly among nonwhites than among whites, narrowing the ratio from 3.8 to 0.6, suggesting improved assignment of cause of death for minority populations.

9. Racial data were not specific for nonwhite groups (eg, black, Asian, American Indian) until recent decades, and racial classification in health statistics, for example in the consistency of coding in numerator and denominator data, remains problematic (7). The specification of blacks among “nonwhite” populations in mortality data only became available in 1970. Nonwhite is an especially heterogeneous category, and it is not clear to whom this category refers. While blacks generally have worse health than whites, Asians generally have better health than whites (8). In order to promote and monitor health equity, it is critical to distinguish among “nonwhite” populations.

References:

1. Linder F, Grove R. Vital statistics rates in the United States, 1900–1940. Washington (DC): US Government Printing Office; 1943.

2. Grove R, Hetzel A. Vital Statistics Rates in the United States, 1940–1960: US Government Printing Office; 1968.

3. American Cancer Society. Cancer Facts & Figures 2018. Atlanta (GA): American Cancer Society; 2018.

4. Faust M, Dolman A. Comparability ratios based on mortality statistics for the fifth and sixth revisions, United States. 1950. Washington (DC): US Department of Health, Education, and Welfare, Public Health Service, National Center for Health Statistics; 1964

5. Anderson RN, Miniño AM, Hoyert DL, Rosenberg HM. Comparability of cause of death between ICD-9 and ICD-10: preliminary estimates. National vital statistics reports 2001;49(2):1–32.

6. Agarwal R, Norton JM, Konty K, Zimmerman R, Glover M, Lekiachvili A, et al. Overreporting of deaths from coronary heart disease in New York City hospitals, 2003. Prev Chronic Dis 2010;7(3):A47. http://www.cdc.gov/pcd/issues/2010/may/09_0086.htm.

7. Hahn RA. The state of federal health statistics on racial and ethnic groups. JAMA 1992;267(2):268–71.

8. Health, United States, 2014: with special feature on adults aged 55–64. Atlanta (GA): Centers for Disease Control and Prevention, National Center for Health Statistics; 2015.
