# Erratum, Vol. 15, March 8 Release

**DOI:** 10.5888/pcd15.170284e

**Published:** 2018-06-14

Because of an author error, references in the appendixes of the article “Trends in Mortality Among Females in the United States, 1900–2010: Progress and Challenges” were incorrectly numbered. The article was corrected on May 30, 2018, and can be found at https://www.cdc.gov/pcd/issues/2018/17_0284.htm. We regret any confusion or inconvenience this error may have caused.

